# Polygenic susceptibility to prostate and breast cancer: implications for personalised screening

**DOI:** 10.1038/bjc.2011.118

**Published:** 2011-04-05

**Authors:** N Pashayan, S W Duffy, S Chowdhury, T Dent, H Burton, D E Neal, D F Easton, R Eeles, P Pharoah

**Affiliations:** 1 Department of Public Health and Primary Care, Institute of Public Health, University of Cambridge, University Forvie Site, Robinson way, Cambridge CB2 0SR, UK; 2 Centre for Cancer Prevention, Wolfson Institute of Preventive Medicine, University of London, London EC1 M 6BQ, UK; 3 PHG Foundation, Cambridge CB1 8RN, UK; 4 Department of Oncology, University of Cambridge, Cambridge CB2 2QQ, UK; 5 Section of Cancer Genetics, The Institute of Cancer Research, Sutton, Surrey SM2 5 NG, UK

**Keywords:** polygenic risk, personalised screening, breast cancer, prostate cancer

## Abstract

**Background:**

We modelled the efficiency of a personalised approach to screening for prostate and breast cancer based on age and polygenic risk-profile compared with the standard approach based on age alone.

**Methods:**

We compared the number of cases potentially detectable by screening in a population undergoing personalised screening with a population undergoing screening based on age alone. Polygenic disease risk was assumed to have a log-normal relative risk distribution predicted for the currently known prostate or breast cancer susceptibility variants (*N*=31 and *N*=18, respectively).

**Results:**

Compared with screening men based on age alone (aged 55–79: 10-year absolute risk ⩾2%), personalised screening of men age 45–79 at the same risk threshold would result in 16% fewer men being eligible for screening at a cost of 3% fewer screen-detectable cases, but with added benefit of detecting additional cases in younger men at high risk. Similarly, compared with screening women based on age alone (aged 47–79: 10-year absolute risk ⩾2.5%), personalised screening of women age 35–79 at the same risk threshold would result in 24% fewer women being eligible for screening at a cost of 14% fewer screen-detectable cases.

**Conclusion:**

Personalised screening approach could improve the efficiency of screening programmes. This has potential implications on informing public health policy on cancer screening.

The benefits of any cancer screening programme may be offset by adverse consequences, such as false-positive findings (positive screening findings that do not result in a diagnosis of cancer), overdiagnosis (diagnosis of a cancer as a result of screening that would not have been diagnosed in a person's lifetime had screening not taken place) (Paci *et al*, 2004), and overtreatment. A screening programme becomes viable if it does more good than harm at reasonable cost (Gray *et al*, 2008).

Prostate and breast cancers are the two most commonly diagnosed cancers in men and women, respectively, in the Western countries (Parkin *et al*, 2005). The value of screening for prostate cancer using serum prostate-specific antigen (PSA) remains controversial even after the publication of the two major randomised controlled trials of screening (Andriole *et al*, 2009; Schroder *et al*, 2009). Early detection of prostate cancer by screening can prevent death for a subset of men, but overdiagnosis and overtreatment may be substantial. The European Study of Screening for Prostate Cancer showed that to prevent one death from prostate cancer, 1410 men would need to be screened and 48 would need treatment (Schroder *et al*, 2009), although more mature data may demonstrate greater effectiveness. In all, 8 out of 1000 men undertaking PSA testing are likely to be overdiagnosed (Pashayan *et al*, 2009). In breast cancer, the benefit of mammographic screening in preventing death is greater than the harm in terms of overdiagnosis. On the basis of the UK Breast Screening Programme, 2.3 out of 1000 women screened for 20 years are likely to be overdiagnosed (Duffy *et al*, 2010).

Genome-wide association studies (GWAS) have identified genetic variants that are common in the population and confer susceptibility to different types of cancers. Most susceptibility variants identified by GWAS in different cancers have low effect size (per-allele relative risks of 1.1–1.3) (Chung *et al*, 2010) and so the clinical utility of the individual variants in predicting future risk is limited. However, the combination of multiple risk alleles, each with a weak effect may result in a distribution of risk in the population that is sufficiently wide to be clinically useful (Pharoah *et al*, 2002).

Several studies have shown that risk-profiles based on the known common susceptibility alleles have limited discrimination for breast cancer (Gail, 2008; Pharoah *et al*, 2008; Wacholder *et al*, 2010) and for prostate cancer (Salinas *et al*, 2009), leading some investigators to conclude that the clinical utility of risk prediction based on polygenic profiling is still limited. However, discrimination is not the only measure of clinical utility of a risk prediction model and it has been suggested that polygenic risk profiling may provide sufficient information to enable screening for breast cancer to be targeted to those women at highest risk (Pharoah *et al*, 2008; Devilee and Rookus, 2010; Wacholder *et al*, 2010). The aim of this study was to model the efficiency of a personalised screening strategy based on a combination of age and polygenic risk-profile compared with a strategy based on age alone in prostate and breast cancer.

## Materials and methods

We compared the number of individuals eligible for screening and the number of cases potentially detectable by screening in the population undergoing screening based on age alone compared with a population undergoing personalised screening based on age and polygenic risk-profile, in which eligibility for screening depends on 10-year absolute risk of being diagnosed with prostate or breast cancer.

### Absolute risk calculation

The number of prostate and breast cancer registrations, deaths from prostate and breast cancer, deaths from all causes, and mid-year population estimates in 1-year age bands for England from 2002 to 2006 were obtained from the Office for National Statistics. These data were used to estimate prostate and breast cancer incidence and mortality rates for prostate cancer, breast cancer, and other causes. We then used the DevCan 6.4.1 software (National Cancer Institute and Information Management Services, 2009) to derive the age-conditional absolute risk (risk between ages x and y, given alive and cancer-free at age x) of being diagnosed with prostate or breast cancer among the general population. DevCan is based on competing risk methods developed by Fay *et al* (2003). We also estimated age-conditional absolute risk for individuals at different levels of polygenic risk by underlying cancer-specific incidence with the polygenic relative risk.

### Polygenic risk distribution

In all, 31 prostate cancer and 18 breast cancer susceptibility loci with common risk alleles have been published (Table 1).

We estimated the variance of the distribution of polygenic risk in the population from the published risk allele frequencies and per-allele relative risk, assuming a log-additive model of interaction between risk alleles both within and between loci. Under this model, the distribution of risk on a relative risk scale in the population at birth is log-normal with mean, *μ*, and variance, *σ*^2^. We set *μ*=−*σ*^2^/2, so that the mean relative risk in the population at birth is equal to unity. The distribution of relative risk among cases at young ages is also log-normal with the same variance, but shifted (on the log scale) to the right by *σ*^2^ (Pharoah *et al*, 2002). The 31 prostate cancer susceptibility variants result in a polygenic variance of 0.377, accounting for approximately 24% of the familial risk of prostate cancer. The 18 breast cancer susceptibility variants confer a polygenic variance of 0.121 and account for approximately 8.4% of the familial risk of breast cancer.

The percentile rank associated with a given polygenic relative risk (or age-conditional absolute risk) in the population or in cases can be calculated given the mean and variance of the log-normal relative risk distribution. We thus estimated the proportion of the population that has a polygenic risk greater than a given absolute risk threshold, and the proportion of cases that will occur within this high-risk subgroup.

We compared two approaches with screening for prostate cancer in men aged 45–79 – screening based on age alone in which men are only eligible for screening from age 55 (10-year absolute risk of 2% or greater), and personalised screening in which men are eligible for screening at a 2% absolute risk that is age and polygenic risk dependent. We then compared the number of individuals eligible for screening under the two approaches and the number of cases occurring in the eligible population that are therefore potentially screen detectable. Similarly, we compared breast cancer screening based on age alone in women aged 47–79 (10-year absolute risk with screening ⩾2.5%) with screening women aged 35–79 with a 2.5% 10-year risk based on age and polygenic profile.

## Results

### Prostate cancer

**Figure 1:**
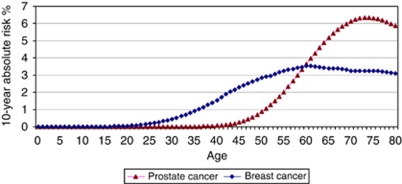
Ten-year absolute risk of being diagnosed with prostate or breast cancer, England, 2002–2006.

On average, there were 22 836 new cases of prostate cancer per year in men 45–79 years in England during the period 2002 to 2006 (total population 8 655 126). The age-conditional absolute risk of being diagnosed with prostate over 10 years in the general population of men in England is shown in Figure 1. Under the age-based screening programme, 63% of men would be eligible for screening (aged 55 and over) and 96% of cases would occur in this subset of the population (Table 2). These are the cases that are potentially screen detectable. Under the personalised strategy, 53% of men would be eligible for screening with 93 of cases being screen detectable. Thus, the number of men eligible for screening would be 17% fewer at a cost of detecting 3% fewer cases. For the population of men aged 45–79 in England, there would be an additional three screen-detectable cases per 100 000 population in men younger than 55 years of age with polygenic risk ⩾2%, and 12 cases per 100 000 population would be missed in men older than 55 years with polygenic risk <2%.

**Figure 2:**
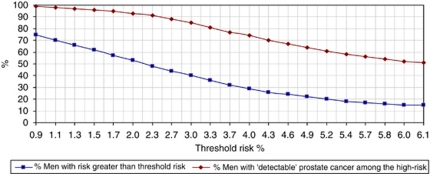
Men eligible for screening and cases detectable by screening at different risk thresholds. Percentage of men 45–79 years of age with polygenic risk for prostate cancer greater than a given threshold risk and percentage of men with detectable prostate cancer within this high-risk population, England, 2002–2006.

The proportion of men 45–79 years that would be eligible for screening and the proportion of cases potentially detectable within the eligible population at different risk thresholds are given in Figure 2.

The eligible population for the personalised approach based on a 1.4% 10-year risk threshold would be the same size as the age 55 and over population. The number of screen-detectable cases would then be 0.4% (one case per 100 000 population) greater under the personalised approach. Alternatively, a 1.5% threshold for personalised screening would be 2.6% (1637 men eligible for screening per 100 000 population) smaller than the age 55 and over population and have the same number of screen-detectable cases. At a higher age threshold, such as a 2.2% threshold for personalised screening, the number eligible for screening would be 4% (1983 per 100 000 population) smaller than the age 58 and over population and have the same number of potentially screen-detectable cases.

Table 2 shows the eligible population and screen-detectable cases for screening from age 51 or an absolute risk threshold of 1%, and screening from age 58 or an absolute risk threshold of 3%.

**Figure 3:**
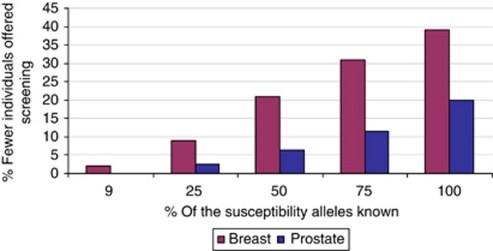
Change in proportion of individuals eligible for screening with increase in the known susceptibility variants. The likely percentage fewer individuals that would be eligible for screening under the personalised screening strategy as compared with the standard screening while detecting the same number of cases with increase in the percentage of the known susceptibility alleles. Prostate: compared with screening men 55–79; currently ∼24% of the variants known. Breast: compared with screening women 47–79; currently ∼9% of the variants known.

If all possible susceptibility variants for prostate cancer were known (predicted polygenic variance 1.58), 35% of men aged 45–79 would be at 2% 10-year risk with 90% of cases being potentially screen detectable. Compared with screening from age 55, 44% fewer men would be offered screening at a cost of 7% fewer cases being potentially screen detectable. To detect the same number of cases as screening from age 55, 20% (12 768 men eligible for screening per 100 000 population) fewer men would be eligible for screening (Figure 3).

### Breast cancer

On average, there were 30 936 new cases of breast cancer per year in women 35–79 years in England during the period 2002–2006 (total population 13 126 890). The age-conditional absolute risk of being diagnosed with breast cancer over 10 years in the general population of women in England is given in Figure 1. Under the age-based programme, 65% of women aged 35–79 would be eligible for screening with 85% of cases being potentially screen detectable (Table 3). Under the personalised strategy, 50% of women would be eligible for screening with 73 of cases being potentially screen detectable. Thus, the number of women eligible for screening would be 24% fewer at a cost of 14% fewer screen-detectable cases. There would be nine screen-detectable cases per 100 000 population under personalised screening in women not eligible under age-based screening and 38 potentially screen-detectable cases per 100 000 population under age-based screening in women not eligible for screening based on polygenic risk (Table 3).

The eligible population for the personalised approach based on a 2.02% 10-year risk threshold would be the same size as the age 47 and over population. The number of screen-detectable cases would then be 1% (two cases per 100 000 population) greater under the personalised approach. Alternatively, a 2% threshold for personalised screening would entail screening 2% fewer women (1477 women eligible for screening per 100 000 population) than the age 47 and over population and yield the same number of potentially screen-detectable cases.

In a best-case scenario analysis, assuming all possible susceptibility variants for breast cancer were known, 28% of women 35–79 years would be at 2.5% risk and 76% of the cases would occur in this group. Compared with screening from age 47, 57% fewer women would be offered screening at a cost of detecting 10% fewer cases. To detect the same number of cases as screening from age 47, 39% (25 678 women eligible for screening per 100 000 population) fewer women would need to be screened (Figure 3).

## Discussion

These data show that personalised screening with eligibility for screening based on an absolute risk that is dependent on age and polygenic risk and equivalent to the risk threshold for eligibility based on age alone could reduce the number of people eligible for screening while detecting the majority of the cancers identified through a programme based on age alone. Alternatively, screening the same number of individuals in a personalised screening programme could potentially detect a greater number of cases than a screening programme based on age alone.

However, we have estimated the proportion of the population to be offered screening and the proportion of cancer cases that might be screen detectable in this subgroup of the population, from the distribution of genetic risk in the population. The estimate of potentially detectable cases is based on cancer incidence derived from cancer registration and is independent of the detection rate by screening. Given the normal distribution of polygenic risk among the cases, and the number of cases in single age group, we estimated the proportion and the expected number of cases that will occur above a certain absolute risk threshold. We have not estimated the expected number of cases to be detected following a screening programme, as this would depend on screening programme sensitivity. Screening programme sensitivity is the probability of detecting cancer by screening in a population subjected to screening. The programme sensitivity increases with decrease in the inter-screening interval, with increase in test sensitivity and with increase in the duration of the pre-clinical screen-detectable phase (Launoy *et al*, 1998). In subjects of a given age at high genetic risk, the test sensitivity is likely to be the same or better than in those of the same age at low genetic risk. However, both the PSA test (Hoffman *et al*, 2002) and mammogram are less sensitive in younger subjects (at lower risk). It is not known how test sensitivity will compare between younger and older subjects at the same absolute risk. The duration of the pre-clinical, screen-detectable phase may also vary by underlying genetic risk. Thus, the comparative sensitivity of the screening programme under the two approaches is not known, and empirical data will be needed in order to estimate this. Assuming equivalent or improved screening programme sensitivity, personalised screening has the potential for cost saving as the cost of the genetic test for risk profiling may be offset by savings on repeat screening and diagnostic work-up of false positives.

Reducing the number of screening tests may also reduce some of the harms associated with screening. Fewer screen tests will, at the population level, reduce the anxiety and inconvenience associated with having the test. Assuming that the probability of a false positive is independent of polygenic risk-profile, reducing the number of screen tests will also reduce the number of false-positive screens, with a reduction in the harms associated with a false positive and the benefit of saving further resources on diagnostic tests. Personalised screening also has the potential to reduce the harms associated with overdiagnosis and overtreatment, but this depends on the nature of the relationship between polygenic risk and disease aggressiveness. To date, there is equivocal evidence on the association of combination of prostate cancer susceptibility variants with disease aggressiveness (Xu *et al*, 2008; Bao *et al*, 2010).

Personalised screening may potentially confer additional benefits. It can detect cancer in younger subjects at high risk. Prostate and breast cancer detected in younger subjects may tend to behave more aggressively (Fredholm *et al*, 2009; Lin *et al*, 2009). If polygenic high risk is associated with disease aggressiveness, then potentially additional life years would be gained by early detection of cancer in younger subjects. The majority of breast cancer susceptibility variants identified to date confer risk for oestrogen receptor-positive breast cancers (Turnbull *et al*, 2010), which are responsive to hormonal treatment and have a favourable prognosis (Dunnwald *et al*, 2007). However, the nature of the complex interaction between disease risk, tumour subtype, natural history of disease, and benefit from screening are not understood and the true benefits of screening according to genetic risk cannot be estimated.

In addition to polygenic risk, there is scope for individualised screening based on phenotypic risk markers. Already, there is considerable screening activity below the age range of the UK national programme for women with a significant family history of breast cancer (Maurice *et al*, 2006). There is also interest in tailoring screening to risk based on mammographic breast density. This might be used to prescribe screening frequency or indeed modality, since in addition to risk, density affects the sensitivity and the potential lead time of mammographic screening (Chiu *et al*, 2010). Further studies are needed using empirical data to test the implications of adding information on PSA test level and family history to polygenic risk profiling for personalised screening in prostate cancer (Zheng *et al*, 2008).

The threshold risk for personalised screening will be population specific. We have used data from England to estimate the proportion of men 45–79 years that would be eligible for screening and the proportion of cases potentially detectable within the eligible population at different risk thresholds. The optimum threshold risk for population of England will be different from that of another population with different incidence of cancer, such as of Asian population with low incidence of prostate cancer.

Other issues need to be considered. Screening based on a personalised risk-profile would add complexity to a screening programme. Perhaps of greater importance is the fact that eligibility for screening based on age is generally acceptable to both professionals and the public, but whether eligibility based on age and other risk factors would also be acceptable is not known. Furthermore, there are ethical and legal issues associated with genetic testing and risk prediction that would need to be addressed before personalised programmes could be implemented.

Personalised screening strategy based on age and genetic risk would potentially improve the efficiency of screening programmes and reduce their adverse consequences. Questions remain whether higher genetic risk affects cancer detection and cancer behaviour and so affecting test sensitivity, overdiagnosis and outcome. Further evidence from empirical data is needed. Nevertheless, this approach has the potential to inform public health policy decision making in the context of population screening.

## Figures and Tables

**Table 1:** Common susceptibility variants for prostate and breast cancer identified through GWAS

**dbSNP No.**	**Locus/gene**	**Risk-allele frequency**	**Odds ratio per allele**	**Variance**	**Reference**
*Prostate*					
rs12621278	2q31/ITGA6	0.94	1.30	0.008	Eeles *et al* (2009)
rs721048	2p15	0.19	1.15	0.002	Gudmundsson *et al* (2008)
rs1465618	2p21/THADA	0.23	1.08	0.002	Eeles *et al* (2009)
rs2660753	3p12	0.11	1.18	0.002	Eeles *et al* (2008)
rs10934853	3q21.3	0.28	1.12	0.002	Gudmundsson *et al* (2009)
rs7679673	4q24 /TET2	0.55	1.09	0.004	Eeles *et al* (2009)
rs17021918	4q22/PDLIM5	0.66	1.10	0.003	Eeles *et al* (2009)
rs12500426	4q22/PDLIM6	0.46	1.08	0.007	Eeles *et al* (2009)
rs9364554	6q25	0.29	1.17	0.013	Eeles *et al* (2008)
rs6465657	7q21	0.46	1.12	0.007	Eeles *et al* (2008)
rs10486567	7p15 /JAZF1	0.77	1.12	0.009	Thomas *et al* (2008)
rs2928679	8p21	0.42	1.05	0.010	Eeles *et al* (2009)
rs1512268	NKX3.1	0.45	1.18	0.014	Eeles *et al* (2009)
rs620861	8q24	0.61	1.28	0.024	Al Olama *et al* (2009)
rs10086908	8q24	0.70	1.25	0.007	Al Olama *et al* (2009)
rs445114	8q24	0.64	1.14	0.041	Gudmundsson *et al* (2009)
rs16902094	8q24	0.15	1.21	0.015	Gudmundsson *et al* (2009)
rs6983267	8q24	0.50	1.26	0.010	Yeager *et al* (2007)
rs1447295	8q24	0.10	1.62	0.004	Amundadottir *et al* (2006)
rs16901979	8q24	0.03	2.10	0.002	Gudmundsson *et al* (2007a)
rs4962416	10q26 /CTBP2	0.27	1.17	0.013	Thomas *et al* (2008)
rs10993994	10q11/MSMB	0.24	1.25	0.014	Eeles *et al* (2008), Thomas *et al* (2008)
rs7127900	11p15	0.20	1.22	0.011	Eeles *et al* (2009)
rs7931342	11q13	0.51	1.16	0.012	Eeles *et al* (2008), Thomas *et al* (2008)
rs4430796	17q12 /HNF1B	0.49	1.24	0.015	Gudmundsson *et al* (2007b)
rs11649743	HNF1B	0.80	1.28	0.015	Sun *et al* (2008)
rs1859962	17q24.3	0.46	1.24	0.017	Gudmundsson *et al* (2007b)
rs2735839	19q13/KLK2,KLK3	0.85	1.20	0.001	Eeles *et al* (2008)
rs8102476	19q13.2	0.54	1.12	0.011	Gudmundsson *et al* (2009)
rs5759167	22q13	0.53	1.16	0.015	Eeles *et al* (2009)
rs5945619	Xp11	0.28	1.12	0.002	Eeles *et al* (2008), Gudmundsson *et al* (2008)
					
*Breast*					
rs11249433	1p11.2	0.39	1.16	0.010	Thomas *et al* (2009)
rs1045485	2q33 /CASP8	0.85	1.14	0.004	Cox *et al* (2007)
rs13387042	2q35	0.49	1.12	0.006	Milne *et al* (2009)
rs4973768	3p24 /NEK10, SLC4A7	0.46	1.11	0.005	Ahmed *et al* (2009)
rs889312	5q11/MAP3K1	0.28	1.13	0.006	Easton *et al* (2007)
rs4415084	5p12/MRPS30	0.40	1.19	0.015	Stacey *et al* (2008)
rs2046210	6p12/ESR1	0.36	1.29	0.030	Zheng *et al* (2009)
rs13281615	8q24	0.40	1.08	0.003	Easton *et al* (2007)
rs1011970	9	0.17	1.09	0.002	Turnbull *et al* (2010)
rs2981582	10q26/FGFR2	0.38	1.26	0.025	Udler *et al* (2009)
rs2380205	10p15	0.43	0.94	0.002	Turnbull *et al* (2010)
rs10995190	10q21/ZNF365	0.85	1.16	0.006	Turnbull *et al* (2010)
rs704010	10q22	0.39	1.07	0.002	Turnbull *et al* (2010)
rs614367	11q13	0.15	1.15	0.005	Turnbull *et al* (2010)
rs3817198	11p15/LSP1	0.30	1.07	0.002	Easton *et al* (2007)
rs999737	14q24/RAD51L1	0.76	1.06	0.001	Thomas *et al* (2009)
rs1244362	16q12/TOX3	0.25	1.20	0.014	Easton *et al* (2007), Stacey *et al* (2007)
rs6504950	17q/COX11	0.73	1.05	0.001	Ahmed *et al* (2009)

Abbreviations: dbSNP=Single Nucleotide Polymorphism database; GWAS=genome-wide association study. Reported risk allele frequency in Europeans.

**Table 2:** Reclassification of population of 100 000 men 45–79 years eligible for screening and in whom prostate cancer could be detectable, under age-based or personalised screening strategies

**Personalised screening**	**Age-based screening**		
**Polygenic risk threshold**	**<51 years**	**⩾51 years**	**Total**
*Population*			
<1%	20 355	9377	29 733
⩾1%	2079	68 188	70 267
Total	22 434	77 566	100 000
			
*Cases*			
<1%	2	3	5
⩾1%	1	258	259
Total	3	261	264
			
**Polygenic risk threshold**	**<55 years**	**⩾55 years**	**Total**
*Population*			
<2%	33 802	13 328	47 130
⩾2%	2841	50 029	52 871
Total	36 643	63 357	100 000
			
*Cases*			
<2%	6	12	18
⩾2%	3	243	246
Total	9	255	264
			
**Polygenic risk threshold**	**<58 years**	**⩾58 years**	**Total**
*Population*			
<3%	46 499	16 152	60 408
⩾3%	4993	35 960	39 592
Total	51 492	52 113	100 000
			
*Cases*			
<3%	15	26	41
⩾3%	7	216	223
Total	22	242	264

Eligibility based on age or polygenic risk equivalent to 10-year absolute for that age considering three scenarios: age 51 *vs* risk 1%, age 55 *vs* risk 2%, age 58 *vs* risk 3% England 2002–2006.

**Table 3:** Reclassification of population of 100 00 women 35–79 years eligible for screening and in whom breast cancer could be detectable, under age-based or personalised screening strategies.

**Personalised screening**	**Age-based screening**		
**Polygenic risk threshold**	**<47 years**	**⩾47 years**	**Total**
*Population*			
<2.5%	30 276	19 926	50 202
⩾2.5%	4429	45 368	49 798
Total	34 705	65 295	100 000
			
*Cases*			
<2.5%	26	38	64
⩾2.5%	9	162	172
Total	35	200	236

Eligibility based on age 47 or polygenic risk equivalent to 10-year absolute risk for age 47 (2.5% 10-year absolute risk); England 2002–2006.
